# Sagittal Realignment Following Decompression for Lumbar Spinal Stenosis in Elderly Patients: A Comprehensive EOS Imaging Analysis

**DOI:** 10.3390/diagnostics14212380

**Published:** 2024-10-25

**Authors:** Hyung-Youl Park, Ho-Young Jung, Geon-U Kim, Se-Heon Lee, Jun-Seok Lee

**Affiliations:** Department of Orthopedic Surgery, Eunpyeong St. Mary’s Hospital, College of Medicine, The Catholic University of Korea, Seoul 03312, Republic of Korea; matrixbest@naver.com (H.-Y.P.);

**Keywords:** decompression, lumbar spinal stenosis, sagittal alignment, EOS imaging system

## Abstract

Background/Objectives: This study investigated whether decompression surgery for lumbar spinal stenosis can effectively improve sagittal alignment in elderly patients. With the growing focus on sagittal balance in spinal surgery, this study aimed to evaluate post-decompression alignment changes and identify the factors influencing these changes using the EOS imaging system. Methods: A retrospective analysis was conducted on 49 elderly patients who underwent decompression surgery alone for lumbar spinal stenosis. Radiologic parameters, measured using the EOS system, and clinical outcomes were assessed preoperatively, at two weeks postoperatively, and at one year postoperatively. Patients were grouped based on the improvement of the sagittal vertical axis (SVA) by 25 mm or more. A multivariate analysis was performed to identify factors affecting sagittal alignment changes. Results: Significant sagittal alignment improvements were observed postoperatively, including a notable increase in thoracic kyphosis and a decrease in SVA observed at one year. Clinical outcomes, such as the Oswestry disability index (ODI) and EQ-5D, significantly improved at both two weeks and one year postoperatively compared to preoperative values (all *p*-values < 0.05). Multivariate analysis revealed that greater preoperative SVA and higher ODI were significant predictors of sagittal alignment changes (odds ratio [OR] for SVA = 1.014, OR for ODI = 1.034). Conclusions: Decompression surgery for lumbar spinal stenosis in elderly patients can result in significant improvements in sagittal alignment and clinical outcomes. The study suggests that decompression alone is a viable surgical option for elderly patients, particularly those with a greater preoperative sagittal imbalance and disability, even in the absence of major deformities.

## 1. Introduction

With advancements in medical treatments and an increasing life expectancy, the prevalence of spinal disorders in elderly patients is rising, necessitating proper management to maintain a better quality of life [1]. Recent studies have suggested that sagittal alignment is more closely associated with pain and disability compared to other spinal parameters [2,3]. Moreover, sagittal compensation for the standing posture correlated with health-related quality of life measurements [4]. In elderly patients, sagittal malalignment is prevalent, making the restoration of sagittal balance an essential consideration when planning spinal surgery [5].

Sagittal imbalance in older patients can come from various causes, including disc and bone degeneration, traumatic kyphosis following compression fractures, and adult spinal deformities such as lumbar degenerative kyphosis [6]. However, reconstructive surgeries for these conditions are complex, often posing high risks for elderly patients due to comorbidities and osteoporosis, which increase the rates of complications and reoperations [7,8].

However, extensive surgery is not always necessary. Lumbar spinal stenosis is commonly observed in the elderly, and surgical intervention rates are increasing due to its high prevalence [1,9]. Neurogenic claudication caused by spinal canal narrowing is a typical presentation, often relieved by forward bending. Sagittal malalignment in these patients may result from sustained forward leaning to alleviate neurogenic claudication [1,10]. This intentional sagittal imbalance may be improved with simple decompression surgeries, avoiding the need for more complex reconstructive procedures [11,12]. Improving sagittal alignment through decompression alone offers several benefits, including enhanced posture, mobility, and functional recovery. Moreover, by addressing sagittal malalignment, both neurological symptoms and long-term quality of life are improved [13].

This study aimed to assess whether decompression surgery for lumbar spinal stenosis can improve sagittal alignment in elderly patients using the EOS system (https://www.eos-imaging.com and the accessed on 1 March 2024). Patients included in this study underwent decompression surgery for central lumbar stenosis based on MRI classification. Exclusion criteria were patients requiring fusion surgery, prior spinal surgery, follow-up less than one year, or significant adult deformities. Additionally, this study evaluated preoperative factors affecting global sagittal alignment to determine the suitability of decompression surgery alone versus more complex deformity correction procedures.

## 2. Materials and Methods

Radiologic and clinical data from patients treated at a university-affiliated hospital between January 2020 and December 2020 were retrospectively reviewed. Patients who underwent decompression surgery alone for central stenosis with concomitant lumbar disc herniation, regardless of the number of levels affected, were included. Based on Lee’s magnetic resonance imaging (MRI)-based classification, patients with grade two or three for central stenosis were eligible [14,15]. Patients with moderate or severe foraminal stenosis or dynamic instability, requiring fusion surgery, were not included in this study [10]. Other exclusion criteria included a history of previous spinal surgery, follow-up periods of less than one year, incomplete clinical data, and significant adult spinal deformities such as scoliosis greater than 10 degrees and lumbar degenerative kyphosis with typical cardinal signs [7,16]. The institutional review board approved the study protocol (PC21RASI0018), and the requirement for informed consent was waived due to the retrospective nature of this study.

### 2.1. EOS^®^ Imaging System

**Figure 1:**
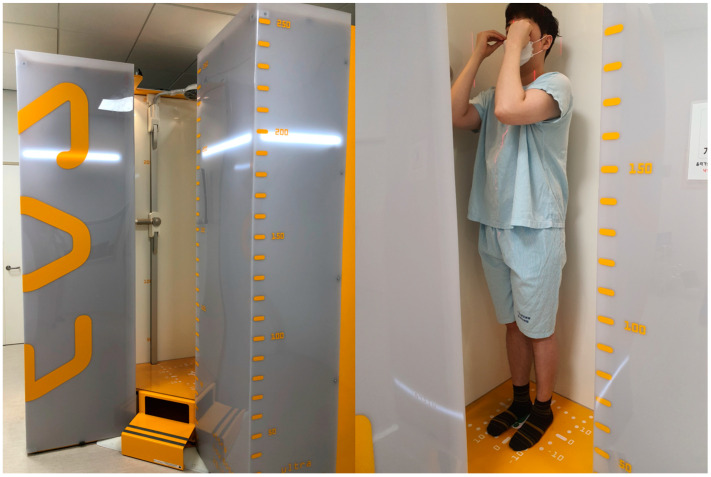
The EOS^®^ imaging system comprises two pairs of X-ray tubes and detectors positioned at 90 degrees to each other. Patients are asked to stand comfortably with their feet side by side, knees straight, and fingertips covering their eyes to prevent the vertebral body from being obscured.

EOS® imaging system (EOS Imaging, Paris, France)is a low-dose biplanar radiography system that has been commercially available since 2007 [17]. This system uses two pairs of X-ray tubes and detectors placed at 90 degrees to each other. EOS^®^ provides several advantages, including reduced radiation exposure compared to digital radiography, 3D reconstructions, whole-body imaging in a functional standing position, high reproducibility in alignment measurements, and faster imaging times [17]. Patients are asked to stand comfortably with their feet side by side, knees straight, and fingertips covering their eyes to avoid obscuring the vertebral body (Figure 1) [18,19].

**Figure 2:**
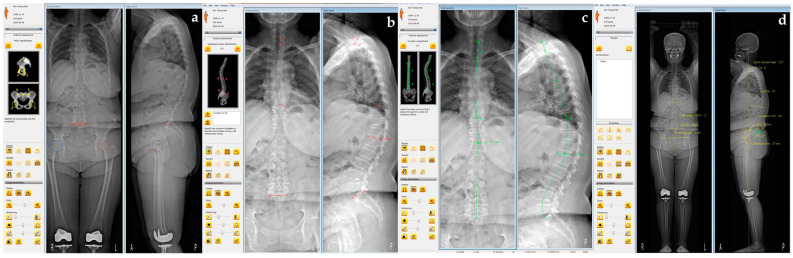
Three-dimensional reconstruction of images using SterEOS^®^ software: (**a**) After standardizing the radiograph to align the femoral heads in the lateral view, the positions of the T1 and S1 superior endplates are identified. (**b**) The software generates a line that can be manually adjusted according to the spinal alignment and the center of the vertebral column. (**c**) Each vertebra’s morphology is defined by identifying 28 points. (**d**) Finally, a 3D spine model is reconstructed using a database of vertebral 3D morphology.

The 3D reconstructions are generated using SterEOS® software (version 1.10), which processes two simultaneously captured orthogonal images. This software employs bone shape recognition algorithms and parametric modeling to create detailed 3D images (Figure 2) [20,21]. To assess inter-observer reliability, two radiologists independently created 3D reconstructions.

### 2.2. Radiologic and Clinical Parameters

Radiologic parameters included spinopelvic parameters such as pelvic incidence (PI), pelvic tilt (PT), and sacral slope (SS). Global sagittal parameters, including lumbar lordosis (LL), thoracic kyphosis (TK), PI-LL mismatch, and sagittal vertical axis (SVA), were assessed using EOS imaging system images taken preoperatively, two weeks postoperatively, and one year postoperatively [7,22].

Clinical outcomes included patient-reported outcome measures such as the 10-point numeric rating scale (NRS) for back and leg pain, the Oswestry disability index (ODI), and the EQ-5D to assess health-related quality of life, evaluated at the same time points [23].

### 2.3. Identifying Factors for SVA Improvement

Two groups were established based on an SVA improvement of 25 mm, which was the average change observed at the one-year follow-up. The SVA improvement group included patients with a decrease in SVA of 25 mm or more, while the non-SVA improvement group consisted of those with less than a 25 mm decrease. Patient demographics and radiologic and clinical outcomes were compared between the two groups, and multivariate analysis was conducted to identify factors associated with SVA improvement.

### 2.4. Statistics

Perioperative continuous variables were presented as means and standard deviations. A paired *t*-test or Wilcoxon signed-rank test was used to compare radiologic and clinical outcomes between the preoperative and postoperative periods. For comparisons between the SVA improvement groups, a Student’s *t*-test or Mann–Whitney test was applied based on the results of the Kolmogorov–Smirnov test. Categorical variables were compared using Pearson’s chi-square test or Fisher’s exact test, depending on the sample size. Inter-observer reliability was measured using the intraclass correlation coefficient (ICC). A multivariate logistic regression was performed to identify independent factors affecting SVA improvement [24]. All statistical analyses were conducted using SPSS software (IBM SPSS Statistics, Version 26.0, Armonk, NY, USA), with significance set at *p* < 0.05.

## 3. Results

### 3.1. Patient Demographics

This study included 49 patients who underwent decompression alone for lumbar spinal stenosis. The mean age was 69.7 ± 10.6 years, with 30 female and 19 male patients. Of these, 11 patients (22.4%) had diabetes, and 31 (63.3%) had hypertension. The American Society of Anesthesiologists (ASA) classification was Grade 2 for 35 patients, Grade 1 for 11 patients, and Grade 3 for 3 patients. Six patients (12.2%) were current smokers. The mean T-scores at the spine and hip were −0.7 ± 1.6 and −1.4 ± 1.3, respectively. The average operation time was 125.8 ± 34.7 min, with a mean intraoperative blood loss of 164.7 ± 170.8 mL. Two patients required a transfusion, with a mean volume of 230 mL. Six patients (12.2%) were admitted to the intensive care unit (ICU). In total, 86 levels were decompressed, with an average of 1.8 ± 0.8 levels per patient. The most frequently treated level was L4-5 (50.0%), followed by L3-4 (24.4%), L5-S1 (16.3%), and L1-2/L2-3 (9.3%) (Table 1).

### 3.2. Radiologic and Clinical Outcomes

Radiologic and clinical outcomes are presented in Table 2. ICC for PI, a constant radiologic parameter, was 0.927, and for all parameters combined, the ICC was 0.958, indicating excellent inter-observer reliability. Spinopelvic parameters such as PI, PT, and SS showed no significant differences between the preoperative, two-week postoperative, and one-year postoperative assessments. However, TK (T4/T12) increased significantly at one year (29.3 ± 11.9 vs. 31.4 ± 12.3, *p* = 0.003), and PI-LL mismatch significantly decreased (13.0 ± 14.7 vs. 10.3 ± 15.1, *p* = 0.014). Additionally, SVA showed a significant decrease at both two weeks and one year (49.4 ± 51.1 vs. 31.2 ± 45.1 vs. 24.5 ± 43.8, *p* < 0.05).

In terms of clinical outcomes, NRS scores for back and leg pain improved postoperatively and remained improved at one year (5.7 ± 3.2 vs. 2.9 ± 2.4 vs. 2.7 ± 2.3 for back pain; 7.3 ± 2.5 vs. 3.2 ± 2.4 vs. 3.0 ± 2.8 for leg pain; all *p* < 0.001). Additionally, quality of life improved, as demonstrated by significant reductions in ODI and improvements in EQ-5D at both two weeks and one year postoperatively (49.6 ± 21.2 vs. 35.3 ± 20.3 vs. 26.0 ± 17.9 for ODI; 2.0 ± 0.4 vs. 1.6 ± 0.3 for EQ-5D, all *p* < 0.001).

### 3.3. Patient Demographics According to SVA Improvement

Based on a 25 mm improvement in SVA, 20 patients were categorized into the SVA improvement group, while 29 were placed in the SVA non-improvement group (Table 3). There were no significant differences in age, gender distribution, prevalence of diabetes, hypertension, or ASA classification between the two groups (all *p*-values > 0.05). Both groups also showed similar proportions of current smokers and bone mineral density values at the spine and hip. Surgical parameters, such as average operation time (125.9 ± 31.1 vs. 125.7 ± 37.6 min, *p* = 0.984), intraoperative blood loss (164.0 ± 140.6 vs. 165.2 ± 191.3 mL, *p* = 0.981), and ICU admission rates (10% vs. 13.8%, *p* = 1.000), were not significantly different between the groups. The average number of operated levels was 1.8 ± 0.9 in the SVA improvement group and 1.6 ± 0.7 in the non-improvement group. The most commonly operated level was L4-5 (54.5% vs. 45.3%), followed by L3-4 (27.3% vs. 24.5%) and L5-S1 (12.1% vs. 18.9%).

### 3.4. Radiologic and Clinical Outcomes According to SVA Improvement

Radiologic and clinical outcomes by SVA improvement are presented in Table 4. Regarding radiologic outcomes, global sagittal alignment parameters such as TK, LL, and SVA were compared between the groups. Preoperative SVA was significantly higher in the SVA improvement group compared to the non-improvement group (68.7 ± 46.6 vs. 36.1 ± 50.5 mm, *p* = 0.027). At two weeks postoperatively, global sagittal alignment parameters were similar in both groups. However, at one year, changes in LL and SVA were more significant in the SVA improvement group than in the non-improvement group (6.9 ± 7.6 vs. −1.9 ± 6.1°, *p* < 0.001 for LL; 10.5 ± 28.2 vs. 34.2 ± 50.1 mm, *p* = 0.04 for SVA).

Regarding clinical outcomes, preoperative ODI was significantly greater in the improvement group compared to the non-improvement group (57.5 ± 19.2 vs. 44.1 ± 21.0%, *p* = 0.028). Preoperative NRS scores for back and leg pain, as well as EQ-5D scores, were similar between the two groups. Although all clinical outcomes improved at two weeks and one year postoperatively, there were no significant differences between the two groups in terms of preoperative outcomes (all *p*-values > 0.05).

### 3.5. Risk Factors Affecting SVA Improvement

A multivariate logistic regression analysis was conducted to identify factors affecting SVA improvement, including preoperative radiologic and clinical parameters (Table 5). Patient-related factors, such as age, sex, diabetes, and hypertension, were not found to be significant risk factors. However, preoperative SVA was a significant radiologic factor affecting SVA improvement (odds ratio [OR] = 1.014, 95% confidence interval [CI] 1.001–1.027, *p* = 0.035). Among clinical parameters, preoperative ODI was also identified as a significant factor (OR = 1.034, 95% CI 1.002–1.067, *p* = 0.036). Patients with greater preoperative SVA and higher ODI showed a higher likelihood of SVA improvement at the one-year follow-up.

### 3.6. Representative Case

**Figure 3:**
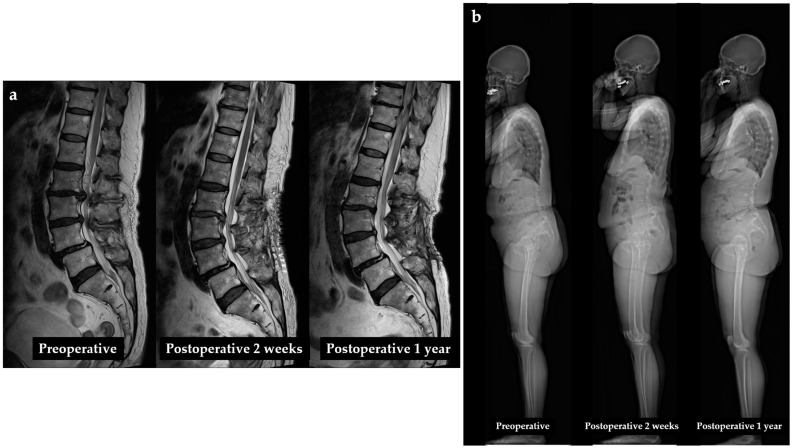
An 80-year-old female patient undergoing decompression for lumbar spinal stenosis: (**a**) magnetic resonance imaging shows lumbar spinal stenosis and decompression of the spinal canal after surgery; (**b**) EOS imaging shows preoperative sagittal imbalance and improvement of sagittal alignment at two weeks and one year postoperatively.

An 80-year-old female patient with underlying dyslipidemia presented with radiating pain in both lower extremities and claudication. Despite receiving medications and epidural steroid injections, her symptoms did not improve. She underwent a three-level decompression at L2-3, L3-4, and L4-5. MRI of the lumbar spine at two weeks and one year postoperatively showed adequate decompression of the spinal canal (Figure 3a). EOS imaging taken at two weeks and one year postoperatively also demonstrated significant improvement in global sagittal alignment compared to preoperative imaging (Figure 3b).

## 4. Discussion

Can decompression for lumbar spinal stenosis improve sagittal alignment in elderly patients? The answer remains somewhat unclear [11]. In this study, we evaluated changes in sagittal parameters after decompression surgery using the EOS system, which offers advantages in accuracy compared to traditional standing radiographs. Whole-spine radiographs are often subject to image distortion, particularly at the edges, and can have low inter-observer reliability [25]. In contrast, the EOS system provides more consistent and reliable measurements, as shown by our high inter-observer reliability for PI (ICC = 0.927). This is consistent with the findings of Shakeri et al. [25], who reported excellent inter-observer reliability, with values ranging from 0.90 to 0.98 for spinopelvic parameters using the EOS system.

Patients with lumbar spinal stenosis often adopt a forward-leaning posture to alleviate neural compression, a compensatory mechanism that reduces pressure on the compressed nerve roots. This sustained forward flexion can lead to a positive SVA and contribute to sagittal malalignment over time. Additionally, compensatory adjustments, such as increased TK and PT, may help maintain balance but can further exacerbate overall sagittal imbalance [1,10]. In our study, spinopelvic parameters like PI, PT, and SS did not show significant changes before and after surgery (all *p*-values > 0.05). However, TK and PI-LL mismatch significantly improved one year postoperatively compared to preoperative values (29.3° vs. 31.4° for TK, 13.0° vs. 10.3° for PI-LL mismatch). Additionally, SVA continuously decreased at both the two-week and one-year follow-ups (49.4 vs. 31.2 vs. 24.5 mm). These results are consistent with prior studies [26,27]. Bouknaitir et al. [26] investigated changes in sagittal alignment in 42 patients who underwent decompression alone for lumbar spinal stenosis without significant deformity over a six-month follow-up. Sagittal balance significantly improved, with SVA and PI-LL mismatch decreasing (52.3 mm vs. 33.9 mm, *p* = 0.0001 for SVA; 8.4° vs. 5.8°, *p* = 0.002 for PI-LL mismatch). A meta-analysis on the effect of lumbar laminectomy on spinal sagittal alignment also reported pooled effect sizes of 3.0° for LL, −1.6° for PT, and −9.6 mm for SVA, respectively [27].

This suggests that decompression surgery can result in meaningful sagittal realignment over time, with the potential for long-term improvements beyond the one-year period. Additionally, the severity of lumbar spinal stenosis could be associated with preoperative sagittal malalignment. Trenchfield et al. [28] demonstrated that patients with severe stenosis had significantly lower LL and SS, as well as higher PI-LL mismatch preoperatively. However, the extent of sagittal alignment changes after surgery was similar in both severe and non-severe cases. All clinical outcomes, including NRS scores for back and leg pain, ODI, and EQ-5D, showed significant improvement at both two weeks and one year postoperatively. Previous studies have similarly reported that patient-reported outcomes significantly improve following spinal decompression surgery. In particular, the correlation between SVA and clinical outcomes was statistically significant at both preoperative and postoperative time points [26]. In this study, greater preoperative SVA and higher ODI were observed in the SVA improvement group. However, a smaller SVA at one year postoperatively was not associated with a significantly better ODI, likely due to the substantial ODI improvement seen in both groups (25.0% vs. 26.8%). A previous study has demonstrated that SVA improvement after decompression surgery is associated with better clinical outcomes, especially for patients with preoperative sagittal malalignment, who tend to show significant improvements in lower-back pain and functional scores at short- and long-term follow-ups [13].

What are the ideal indications for improving sagittal alignment following decompression alone for lumbar spinal stenosis? In this study, we compared radiologic and clinical parameters between the SVA improvement and non-improvement groups, based on an SVA decrease of more than 25 mm, which was the average SVA change at the one-year follow-up. Both patient and surgical parameters were similar in both groups. However, preoperative SVA (68.7 vs. 36.1 mm), changes in LL (6.9° vs. −1.9°), and SVA (10.5 vs. 34.2 mm) at one year postoperatively were significantly different between the two groups. Furthermore, preoperative ODI in the SVA improvement group was significantly higher than in the non-improvement group (57.5% vs. 44.1%). Multivariate analysis using logistic regression indicated that a higher preoperative SVA (OR = 1.014) and ODI (OR = 1.034) were predictive of greater sagittal improvement after decompression surgery.

In a systematic review of spontaneous correction of sagittal malalignment after decompression surgery without corrective fusion, Ogura et al. [11] found that a preoperative PI-LL mismatch of more than 20° and a preoperative SVA of more than 80 mm were strong predictors for postoperative sagittal imbalance. While our study shows that patients with greater preoperative SVA had a higher likelihood of sagittal alignment improvement, the ideal indication for surgery may be limited to cases with a preoperative PI-LL mismatch of less than 20° and SVA of less than 80 mm. Higher preoperative ODI values, which reflect the severity of neurologic claudication, are strongly associated with sagittal imbalance in patients with lumbar spinal stenosis, and greater ODI scores are correlated with increased potential for postoperative sagittal alignment improvement due to more severe functional impairment and imbalance before surgery [27].

The clinical implications of this study suggest that sagittal malalignment can result from claudication and symptoms associated with lumbar spinal stenosis. Neural decompression relieves these symptoms and reduces forward bending, leading to some degree of sagittal alignment improvement [26]. While significant improvements in SVA were observed in our study, there was no corresponding change in LL. This suggests that the clinical improvements were primarily related to global sagittal alignment rather than LL, which is typically addressed in fusion surgeries. Although decompression surgery alone may improve global sagittal parameters through symptom relief, correcting sagittal imbalance with spinopelvic mismatch may require more complex deformity surgeries [11,26]. However, deformity correction in elderly patients should be considered carefully due to the risks associated with underlying comorbidities and the high potential for complications [7]. Minimally invasive techniques, such as endoscopic surgery, may help reduce complications, particularly in elderly patients [29,30].

This study has several limitations. First, as a retrospective cohort study, it is subject to selection bias and incomplete data. While our institution routinely performed preoperative EOS imaging on spinal surgery patients, the retrospective design limited the collection of detailed information on patients’ physical activity levels and occupations, factors that may have affected sagittal alignment outcomes. Second, the number of included patients was relatively small, although 49 exceeded the 28 needed to compare matched pairs. Third, the follow-up period was limited to one year, and long-term results may differ from those of this study. A previous study has shown that sagittal balance and clinical outcomes may continue to improve over a longer follow-up period [26]. Therefore, future prospective studies with larger samples are needed to validate our findings. Despite these limitations, a key strength of this study is its evaluation of sagittal alignment using the EOS system in elderly patients without adult spinal deformity, who underwent decompression alone for lumbar spinal stenosis.

## 5. Conclusions

Decompression surgery for lumbar spinal stenosis can significantly improve global sagittal alignment, including TK, PI-LL mismatch, and SVA, at one year postoperatively. Additionally, patient-reported clinical outcomes showed significant improvements at both two weeks and one year. In the SVA improvement group, patients with greater preoperative SVA experienced significant changes in LL and SVA compared to the non-improvement group. Multivariate analysis identified preoperative SVA and ODI as independent predictors of sagittal alignment improvement. This study suggests that decompression alone can be successful as an option for improving sagittal alignment in elderly patients with stenosis-related imbalance.

## Figures and Tables

**Table 1:** Patient demographics included in this study.

	Decompression Alone	
Total number of patients	49	
Age	69.7 ± 10.6	
Male:female	19:30	
DM	11 (22.4%)	
HTN	31 (63.3%)	
ASA classification (1:2:3)	11:35:3	
Current smoking	6 (12.2%)	
T-score (spine)	−0.7 ± 1.6	
T-score (hip)	−1.4 ± 1.3	
Operation time (minutes)	125.8 ± 34.7	
Intraoperative bleeding (mL)	164.7 ± 170.8	
Transfusion	2 (4.1%)	
ICU admission	6 (12.2%)	
Total levels	1.8 ± 0.8	
Treated level	L1-2	2 (2.3%)
	L2-3	6 (7.0%)
	L3-4	21 (24.4%)
	L4-5	43 (50.0%)
	L5-S1	14 (16.3%)
	Total	86 (100%)

Note: DM—diabetes mellitus, HTN—hypertension, ASA—American Society of Anesthesiologists, ICU—intensive care unit.

**Table 2:** Radiographic and clinical outcomes at three time points: preoperative, postoperative two weeks, and postoperative one year.

	Preoperative	Postoperative Two Weeks	*p*-Value ^1^	Postoperative One Year	*p*-Value ^2^
Radiologic					
PI	54.8 ± 12.3	54.8 ± 11.3	0.934	53.8 ± 11.1	0.066
PT	19.6 ± 10.2	18.9 ± 10.0	0.319	18.9 ± 9.8	0.395
SS	35.1 ± 8.2	36.0 ± 8.8	0.222	34.8 ± 9.0	0.708
T4/T12 kyphosis	29.3 ± 11.9	30.5 ± 11.8	0.078	31.4 ± 12.3	**0.003**
L1/S1 lordosis	41.8 ± 15.2	43.6 ± 14.4	0.096	43.5 ± 15.3	0.149
PI-LL	13.0 ± 14.7	11.1 ± 14.3	0.114	10.3±15.1	**0.014**
SVA	49.4 ± 51.1	31.2 ± 45.1	**0.005**	24.5 ± 43.8	**<0.001**
Clinical					
NRS (back)	5.7 ± 3.2	2.9 ± 2.4	**<0.001**	2.7 ± 2.3	**<0.001**
NRS (leg)	7.3 ± 2.5	3.2 ± 2.4	**<0.001**	3.0 ± 2.8	**<0.001**
ODI	49.6 ± 21.2	35.3 ± 20.3	**<0.001**	26.0 ± 17.9	**<0.001**
EQ-5D	2.0 ± 0.4	1.6 ± 0.3	**<0.001**	1.5 ± 0.4	**<0.001**

Note: PI—pelvic incidence, PT—pelvic tilt, SS—sacral slope, LL—lumbar lordosis, SVA—sagittal vertical axis, NRS—numeric rating scale, ODI—Oswestry disability index. ^1^ Comparison between the preoperative and postoperative two-week periods. ^2^ Comparison between the preoperative and postoperative one-year periods.

**Table 3:** Patient demographics according to SVA improvement ^1^.

	SVA Improvement	SVA Non-Improvement	*p*-Value
Number of patients	20	29	
Age	68.3 ± 10.9	70.6 ± 10.5	0.458
Male:Female	9:11	10:19	0.458
DM	2 (10%)	9 (31.0%)	0.162
HTN	12 (38.7%)	19 (65.5%)	0.694
ASA classification (1:2:3)	6:12:2	5:23:1	0.678
Current smoking	3 (15.0%)	3 (10.3%)	0.677
T-score (spine)	−0.3 ± 1.9	−0.9 ± 1.3	0.253
T-score (hip)	−1.3 ± 1.4	−1.5 ± 1.0	0.591
Operation time (min)	125.9 ± 31.1	125.7 ± 37.6	0.984
Intraoperative bleeding (mL)	164.0 ± 140.6	165.2 ± 191.3	0.981
ICU admission	2 (10%)	4 (13.8%)	1.000
Total levels	1.8 ± 0.9	1.6 ± 0.7	0.354
Treated levels			
L1-2	0 (0%)	2 (3.8%)	>0.05
L2-3	2 (6.1%)	4 (7.5%)	
L3-4	9 (27.3%)	13 (24.5%)	
L4-5	18 (54.5%)	24 (45.3%)	
L5-S1	4 (12.1%)	10 (18.9%)	
Total	33 (100%)	53 (100%)	

Note: SVA—sagittal vertical axis, DM—diabetes mellitus, HTN—hypertension, ASA—American Society of Anesthesiologists, ICU—intensive care unit. ^1^ SVA improvement means a decrease of more than 25 mm on average at the one-year follow-up in the SVA.

**Table 4:** Radiographic and clinical outcomes according to SVA improvement.

	SVA Improvement	SVA Non-Improvement	*p*-Value
Number of patients	20	29	
Radiological outcomes			
Preoperative			
TK	29.6 ± 14.6	29.2 ± 10.0	0.922
LL	37.2 ± 16.2	45.0 ± 13.9	0.074
SVA	68.7 ± 46.6	36.1 ± 50.5	**0.027**
Postoperative two weeks			
TK	31.8 ± 14.5	29.7 ± 10.7	0.548
LL	43.3 ± 15.4	43.9 ± 13.9	0.885
SVA	27.6 ± 39.9	33.8 ± 48.8	0.640
Postoperative one year			
TK	33.1 ± 13.6	30.2 ± 11.4	0.417
LL	37.2 ± 16.2	45.0 ± 13.9	0.074
Change in LL	6.9 ± 7.6	−1.9 ± 6.1	**<0.001**
SVA	10.5 ± 28.2	34.2 ± 50.1	**0.04**
Clinical outcomes			
Preoperative			
NRS (back)	5.8 ± 3.2	5.6 ± 3.3	0.823
NRS (leg)	7.0 ± 2.5	7.5 ± 2.5	0.543
ODI	57.5 ± 19.2	44.1 ± 21.0	**0.028**
EQ-5D	2.1 ± 0.5	1.9 ± 0.3	0.08
Postoperative two weeks			
NRS (back)	2.5 ± 2.0	3.2 ± 2.7	0.326
NRS (leg)	2.8 ± 2.1	3.5 ± 2.5	0.331
ODI	35.1 ± 23.5	35.4 ± 18.2	0.963
EQ-5D	1.6 ± 0.4	1.6 ± 0.3	0.893
Postoperative one year			
NRS (back)	2.6 ± 2.0	2.8 ± 2.6	0.763
NRS (leg)	2.8 ± 2.6	3.1 ± 2.9	0.710
ODI	25.0 ± 18.5	26.8 ± 17.9	0.740
EQ-5D	1.5 ± 0.4	1.5 ± 0.4	0.733

Note: SVA = sagittal vertical axis, TK = thoracic kyphosis, LL = lumbar lordosis, NRS = numeric rating scale, ODI = Oswestry disability index.

**Table 5:** Multivariate analysis for SVA improvement.

	Odds Ratio	95% Confidence Interval	*p*-Value
Age	0.979	0.927–1.034	0.449
Male	0.643	0.200–2.067	0.459
DM	4.050	0.771–21.3	0.098
HTN	1.267	0.390–4.11	0.694
PI	0.974	0.928–1.022	0.289
PT	0.983	0.928–1.041	0.549
SS	0.971	0.905–1.043	0.419
T4/T12 kyphosis	1.002	0.955–1.052	0.920
L1/S1 lordosis	0.965	0.926–1.005	0.083
PI-LL	1.020	0.980–1.061	0.338
SVA	1.014	1.001–1.027	**0.035**
NRS (back)	1.021	0.854–1.221	0.818
NRS (leg)	0.930	0.739–1.170	0.535
ODI	1.034	1.002–1.067	**0.036**
EQ-5D	3.928	0.816–18.9	0.088

Note: SVA—sagittal vertical axis, DM—diabetes mellitus, HTN—hypertension, PI—pelvic incidence, PT—pelvic tilt, SS—sacral slope, LL—lumbar lordosis, NRS—numeric rating scale, ODI—Oswestry disability index.

## Data Availability

Original data will be made available upon reasonable request.

## Abbreviations

MRI	Magnetic resonance imaging
PI	Pelvic incidence
PT	Pelvic tilt
SS	Sacral slope
LL	Lumbar lordosis
TK	Thoracic kyphosis
SVA	Sagittal vertical axis
NRS	Numeric rating scale
ODI	Oswestry disability index
ICC	Intraclass correlation coefficient
DM	Diabetes mellitus
HTN	Hypertension
ASA	American Society of Anesthesiologists
ICU	Intensive care unit
OR	Odds ratio
CI	Confidence interval
